# Guidance on priority setting in health care (GPS-Health): the inclusion of equity criteria not captured by cost-effectiveness analysis

**DOI:** 10.1186/1478-7547-12-18

**Published:** 2014-08-29

**Authors:** Ole F Norheim, Rob Baltussen, Mira Johri, Dan Chisholm, Erik Nord, DanW Brock, Per Carlsson, Richard Cookson, Norman Daniels, Marion Danis, Marc Fleurbaey, Kjell A Johansson, Lydia Kapiriri, Peter Littlejohns, Thomas Mbeeli, Krishna D Rao, Tessa Tan-Torres Edejer, Dan Wikler

**Affiliations:** 1 Department of Global Public Health and Primary Care, University of Bergen, PB 7800, 5020 Bergen, Norway; 2 Nijmegen International Centre for Health Systems Research and Education (NICHE), Radboud University Nijmegen Medical Centre, Nijmegen, The Netherlands; 3 Centre de Recherche du Centre Hospitalier de l’Université de Montréal (CRCHUM), Québec, Canada; 4 Department of Health Systems Financing, World Health Organization, Geneva, Switzerland; 5 Norwegian Institute of Public Health, Oslo, Norway; 6 Harvard Medical School, Harvard University, Cambridge, USA; 7 National Centre for Priority Setting in Health Care, Linköping University, Linköping, Sweden; 8 Centre for Health Economics, University of York, York, UK; 9 Harvard School of Public Health, Harvard University, Cambridge, USA; 10 Section on Ethics and Health Policy, NIH Clinical Centre, Bethesda, USA; 11 Woodrow Wilson School of Public and International Affairs, Princeton University, Princeton, USA; 12 Department of Health, Aging, and Society, McMaster University, Hamilton, Canada; 13 Department of Primary Care and Public Health Sciences, Division of Health and Social Care Research, King’s College London, Previous affiliation the National Institute for Health and Care Excellence (NICE), London, England, UK; 14 Ministry of Health and Social Services, Windhoek, Namibia; 15 Public Health Foundation of India, New Delhi, India

**Keywords:** Priority setting, Resource allocation, Cost-effectiveness, Equity, Population health

## Abstract

This Guidance for Priority Setting in Health Care (GPS-Health), initiated by the World Health Organization, offers a comprehensive map of equity criteria that are relevant to health care priority setting and should be considered in addition to cost-effectiveness analysis. The guidance, in the form of a checklist, is especially targeted at decision makers who set priorities at national and sub-national levels, and those who interpret findings from cost-effectiveness analysis. It is also targeted at researchers conducting cost-effectiveness analysis to improve reporting of their results in the light of these other criteria.

The guidance was develop through a series of expert consultation meetings and involved three steps: i) methods and normative concepts were identified through a systematic review; ii) the review findings were critically assessed in the expert consultation meetings which resulted in a draft checklist of normative criteria; iii) the checklist was validated though an extensive hearing process with input from a range of relevant stakeholders.

The GPS-Health incorporates criteria related to the disease an intervention targets (severity of disease, capacity to benefit, and past health loss); characteristics of social groups an intervention targets (socioeconomic status, area of living, gender; race, ethnicity, religion and sexual orientation); and non-health consequences of an intervention (financial protection, economic productivity, and care for others).

## Introduction

Priority setting of health interventions should seek to achieve health system goals, broadly defined as maximization of health, reduction of inequities in health, and financial protection against the costs of ill health [1,2]. Present methods for priority setting are poorly adapted to address the full range of health system objectives. The main approach to establishing health priority setting, cost-effectiveness analysis, addresses only the first objective of maximising health [3-12]. How governments and other responsible authorities balance health maximization with equity and financial protection has far-reaching implications for what health priorities are agreed and pursued [13]. Three is therefore urgent need for a more explicit recognition of these additional concerns.

The present Guidance for Priority Setting in Health Care (GPS-Health) offers a checklist of equity criteria that are relevant to health care priority setting and are not adequately considered by cost-effectiveness analysis. Decision-makers should carefully consider these criteria alongside results of cost-effectiveness analyses when making decisions on the funding of one intervention and the refusal to fund another.

## Process of guidance development

GPS-Health was develop through a series of expert consultation meetings and involved three steps: i) Methods and equity considerations were identified through a systematic review [14]. This review concluded that several viable techniques to integrate equity concerns within CEA now exist, ranging from descriptive approaches to quantitative methods. Two obstacles at the normative level have impeded their use in decision making to date: the multiplicity of concepts and values discussed under the rubric of equity, and the lack of a widely accepted normative source on which to ground controversial value choices. Clarification of equity concepts and attention to procedural fairness may strengthen use of these techniques in decision-making. ii) The expert group – with representatives from the International Society on Priorities in Health Care, WHO, the National Institute for Health and Clinical Excellence (UK), decision makers from low- and middle income countries, and ethicists and health economists from various academic institutions – met to validate and refine the list of proposed normative criteria. iii) The draft document was presented to the International Society on Priority Setting in Health and other interested stakeholder to improve the recommendations. Input from this hearing process was used in the final revision of the document.

## Target groups and proposed use

The guidance is especially targeted at decision makers who set priorities at national and sub-national levels and who interpret findings from cost-effectiveness analysis. It is intended to be used as a practical checklist that helps them to make sure they have considered all the main issues of equity that are not captured by cost-effectiveness analysis. It is also targeted at researchers conducting cost-effectiveness analysis to improve reporting of their results in the light of these other criteria. Clinicians, and others who want to participate in informed public debate, may also benefit from this guidance.

The guidance is particularly useful if evidence exists on the cost-effectiveness for a wide range of health interventions, and important choices need to be made on which intervention to fund, and for whom. WHO’s CHOICE project has produced a large database of the cost-effectiveness of intervention, which could be one possible starting point for assessing the range or depth of services to be provided [15]. The Disease Control Priorities Project is another source containing relevant cost-effectiveness evidence [16]. Although special attention is given to low- and middle-income countries, this guidance is relevant in all settings [17].

## Principles for priority setting

Priority setting in health is inevitable in all countries around the world. The choices a country makes will always positively or negatively affect some people in the population it serves. Decision makers are therefore accountable to justify their decisions to all those affected and demonstrate that they are aligned with the country’s social values concerning health maximization, health distribution, and financial protection.

Cost effectiveness analysis identifies how resources should be allocated across health interventions so as to maximize health benefits within a given budget, or relative to a threshold level of societal willingness to pay [18]. Concerns about the distribution of health stem from the idea that every person in society should have a fair chance to live a long and healthy life [19]. This principle can be further expressed in terms of vertical equity (requiring the unequal treatment of relevantly unequal cases and pertaining to characteristics of the disease an intervention targets) and horizontal equity (requiring equal treatment of relevantly equal cases and pertaining to characteristics of social groups an intervention targets) [20-22]. The use of either cost-effectiveness analysis or equity analysis for priority setting may lead to different policy recommendations. The key difference is that the former is only concerned about the absolute size of health gains, whereas the latter is concerned about how these health gains are distributed among members of the population. This may lead to striking differences in e.g. provision of health interventions for HIV/AIDS control, in which cost-effectiveness analysis recommends a strategy of universal test and early treatment as an efficient way to reduce the epidemic [23], and equity analysis would suggest prioritizing treatment of severely ill people, mobile treatment services in rural service or food subsidies to poor people [24].

In addition, health interventions also have important consequences outside the health sector, and these may also determine whether certain interventions should be considered priorities. Most importantly, countries may wish to implement interventions that provide financial protection to their populations, in order to reduce poverty associated with ill health or catastrophic health expenditures.

### Criteria for priority setting

The GPS-Health distinguishes three groups of criteria pertaining to i) the disease an intervention targets; ii) the characteristics of the social group an intervention targets; and iii) non-health consequences (Table 1). Below we present rationales for consideration of these criteria in the prioritisation of health interventions, in addition to cost-effectiveness analysis. Several other criteria were also considered, but excluded from the checklist (see Appendix).

**Table 1:** Priority-setting criteria to be considered in conjunction with cost-effectiveness results

**Group 1: disease and intervention criteria**	
Criteria	Question
Severity	Have you considered whether the intervention has special value because of the severity of the health condition (present and future health gap) that the intervention targets?
Realization of potential	Have you considered whether the intervention has more value than the effect size alone suggests on the grounds that it does the best possible for a patient group for whom restoration to full health is not possible?
Past health loss	Have you considered whether the intervention has special value because it targets a group that has suffered significant past health loss (e.g. chronic disability)?
**Group 2: criteria related to characteristics of social groups**	
Criteria	Question
Socioeconomic status	Have you considered whether the intervention has special value because it can reduce disparities in health associated with unfair inequalities in wealth, income or level of education?
Area of living	Have you considered whether the intervention has special value because it can reduce disparities in health associated with area of living?
Gender	Have you considered whether the intervention will reduce disparities in health associated with gender?
Race, ethnicity, religion and sexual orientation	Have you considered whether the intervention may disproportionally affect groups characterized by race, ethnicity, religion, and sexual orientation?
**Group 3: criteria related to protection against the financial and social effects of ill health**	
Criteria	Question
Economic productivity	Have you considered whether the intervention has special value because it enhances welfare to the individual and society by protecting the target population’s productivity?
Care for others	Have you considered whether the intervention has special value because it enhances welfare by protecting the target population’s ability to take care of others?
Catastrophic health expenditures	Have you considered whether the intervention has special value because it reduces catastrophic health expenditures for the target population?

### Group 1: disease-related criteria

For equity reasons, decision-makers may want to attach special value to interventions that target severe health conditions, people with a low capacity to benefit or with large past health losses.

### Severity of health condition

Numerous studies of public preferences show that people put a high value on interventions for particularly severe health conditions, even when they are comparatively less effective than other alternatives [8,25,26]. Many ethical theories also defend giving additional weight to health benefits distributed to those who are worse off in terms of the severity of disease [27-30]. Severe health conditions can refer to a) present health status, b) future health status, or c) future health gap compared with a reference standard of healthy life expectancy. Interventions for such severe conditions are often cost-effective, but not always (see Example 1).

*Example 1*: *Severity of disease and infliximab for fistulising Crohn’s disease in UK*:

In a recent recommendation from NICE on treatment with infliximab for fistulising Crohn’s disease, the incremental cost-effectiveness ratio compared to standard care was found to be €30,300 per QALY gained, which is around, or somewhat higher, than the recommended threshold of approximately €30 000 per QALY gained [31]. In the overall assessment, treatment with infliximab was nevertheless recommended for fistulising Crohn’s because the severity of the condition was considered so high that it justified an adjustment of the threshold typically set: “Although this ICER was considered to be relatively high, the Committee considered the severity of the disease and noted that there were few treatment options available to these patients. The Committee therefore concluded that a planned course of treatment with infliximab for people with fistulising disease could be cost effective if the definition of severe disease was met.” [31].

This example shows that severity of disease sometimes can be seen as an independent criterion for adjusting judgments based on cost-effectiveness only. Other examples of particularly severe health conditions include under-five deaths from infections and malnutrition, maternal deaths, severe maternal complications, chronic schizophrenia, severe bipolar depression, suicide in young age, traffic accidents, HIV, TB, cervical cancer in young age, or severe congenital disorders.

### Realization of potential

From the principle of health maximisation follows that priority should be given to people who benefit most from treatment. However, capacity to benefit is not the only consideration because one may value realization of potentials and wish to offer equal chances to people with different potentials. Many ethicists argue that persons with less treatable health conditions should have just as much of a fair chance to benefit from health care [32,33]. For example, if two potential organ transplant recipients have a potential to benefit that differs, everything else alike, health maximization requires that the person with highest expected benefit should have the organ [34]. By contrast, many argue that equity requires that each be given as much of a fair chance as others (for example through a weighted lottery) to realize their potential. This egalitarian “fair chances” argument has support in the general public and among societal decision makers in several jurisdictions [32,33].

### Past health loss

Concern for past health loss, for example due to chronic disability, is a criterion hitherto less discussed in the literature on priority setting. It derives its justification from a concern for equal lifetime health: all persons should have a fair chance to live a long and healthy life due to the intrinsic importance of health and its instrumental value in enabling fulfilment of life plans [35].

### Group 2: criteria related to characteristics of social groups

Equity in health related to characteristic of social groups has been defined as “the absence of systematic disparities in health between social groups who have different levels of underlying social advantage or disadvantage” [36]. Targeting health interventions to disfavoured groups may be viewed as appropriate when these compensate for underlying factors such as the social determinants of health [4], even when they are not cost-effective. In resource-limited settings, social group factors can be particularly relevant in terms of socio-economic status, area of residence, and gender.

### Socioeconomic status

People with lower socioeconomic status (SES) should have a fair chance to live a full healthy life. Health inequalities related to SES may be unfair when they are the result of an unjust distribution of the socially controllable factors affecting population health [6]. These factors may include social determinants of health, traditional public health measures, and personal medical services. Health interventions to reduce these health inequalities, e.g. food subsidies for people living with HIV/AIDS, hold therefore special value and deserve additional priority (see Example 2) [6,37-40]. However, decision makers should also note that interventions outside the health sector may represent a more effective way to reduce health disparities, such as improving female education (where relevant) or implementing effective regulatory or fiscal measures. Thus, disparities in health associated with non-health determinants of health could have far-reaching implications for judgments about resource allocation outside the health sector.

*Example 2*: *Correcting forsocio-economicinequality in Sweden*:

Swedish politicians responsible for health care in the county councils (n = 449) were asked in a survey about their opinions on trade-offs between interventions that maximizes health vs. interventions that are less efficient but eliminate social inequalities. The scenario assumed that myocardial infarction mortality rate was 50% higher among blue-collar workers than among white collar workers, and that the most cost-effective intervention (i.e. that saved most lives) did not reduce the inequality. The Swedish politicians rejected to only maximise health, and expressed their willingness to sacrifice 15 of 100 preventable deaths to reduce social inequalities [41]. The example illustrates the concept of opportunity cost that is key to all thinking about fair and efficient distribution. The opportunity cost of a health intervention is the value of health forgone if resources are spent elsewhere. The politicians were explicitly thinking about how many extra people would die in order to help a disadvantaged group. Distribution of health benefits between socioeconomic groups matters to decision makers and in this case decision makers rejected allocation strictly according to cost-effectiveness rankings.

### Area of residence

Inequalities in health associated with area of residence are often seen as unacceptable and geographic equity is an often-stated goal in national health policy documents. People living in different areas should all have a fair chance to live a full and healthy life, and this may mean that priority should be given to interventions that target those areas. Localization of clinics for HIV treatment is one example where this criterion is relevant [42]. Because the provision of health care in rural areas, with few people or long travel distances, can be considerably more costly than in urban areas, achieving geographic equity may imply relative loss in overall population health gain [43].

### Gender

In its most general form, men and women should have a fair chance to live a full and healthy life. Since women tend to have a longer life expectancy than men, but lower health status over their lifetime, it is not possible to provide general guidance on whether interventions targeting men or women should be considered a priority. Since there are forms of unfair treatment for women in nearly all countries, however, a special check for gender-related disparities should be considered. This concern is especially relevant for reproductive health services and interventions against domestic violence against women, but also for road traffic injuries among young males and tobacco control for men.

### Race, ethnicity, religion, and sexual orientation

All people, independent of race, ethnicity, religion or sexual orientation, should have a fair chance to live a full and healthy life. This criterion is mainly a non-discriminatory clause, referring to instances in which interventions may be less effective or more costly in certain ethnic groups; prevention of cardiovascular disease among the Maori may be one example. Decision makers should take this into account, and give special value to interventions targeting those groups.

### Group 3: criteria related to protection against the financial and social effects of ill health

This third set of criteria refers to the financial and social protection that some interventions offer [44]. Because of these non-health consequences, these interventions hold special value, and decision makers may sometimes want to prioritize them even if they are not cost-effective.

### Economic productivity

Some health interventions may hold special value as they increase economic productivity more than other interventions, thereby creating additional non-health welfare gains for all through the tax system or other transfer mechanisms [45,46]. Others think that the productivity argument discriminates against the non-productive [47]. Since there is disagreement about these concerns, they are inconsistently incorporated into cost-effectiveness analysis. As a minimum, decision-makers should check whether productivity gains are taken into account, and whether this is not discriminating against the non-productive.

### Care for others

Some health interventions hold special value because they target people in the age where they typically take care of others, e.g. children or elderly. In societies with an extended family structure and less reliance on the state to provide welfare for children or the elderly, welfare for others is widely accepted and relevant to consider.

### Catastrophic health expenditures

Households may be pushed into poverty or forced into deeper poverty because of high out-of-pocket expenditures for health care [1,48]. Protection against catastrophic health expenditures can reduce poverty and improve overall welfare in society [49]. Decision-makers could give priority to public financing of such high-costs interventions, e, g, through subsidised provision or insurance coverage (see Example 3) [50]. In high-income countries with fair financing of health services, this criterion is typically given little weight because insurance coverage is often broad and interventions rarely impose large out-of pocket expenditures [51]. In middle-income countries (and even in some high-income countries), it is often the case that treatment for many serious illnesses is not covered even in these countries. This criterion is therefore relevant and should always be considered.

*Example 3*: *Dialysis to prevent catastrophic health expenditures in Thailand*:

In 2007 Thailand included peritoneal- and hemo-dialysis for patients with end-stage renal disease in their tax-based universal health insurance scheme. The incremental cost-effectiveness ratio (ICER) of initial treatment with peritoneal dialysis was in 2007 estimated to be US$52,000 per quality-adjusted life-year (QALY) gained compared with palliative care [52], and was by many interpreted as not cost-effective. Financial protection was repeatedly used in the policy debate to nevertheless including dialysis in the health insurance scheme: if dialysis for end-stage renal disease would not be covered, it would impose catastrophic health expenditures on patients and thereby push them and their families into poverty.

This example shows that financial protection matters to decision makers and the public, even for cost-ineffective interventions. However, full coverage for all in the universal plan would protect against catastrophic expenditures, but it might also displace services that do much more good. The example shows that the trade-off between maximizing health and financial protection will produce winners and losers, and it is not clear that the health loss from replacing other services that are more cost-effective and fair is well enough justified by financial protection of a few.

## Conclusion

The GPS-Health does not provide a formula or blueprint for equitable priority setting of health interventions but should serve as input to priority setting processes [53]. Decision-makers should consider the checklist in conjunction with cost-effectiveness analysis, and carefully consider the criteria they find relevant to their health system and political context.

Since efficiency, equity and financial protection are considerations that sometimes conflict with each other, decision makers need to weigh them against each other and make trade-offs. It should be recognized that, in a resource-constrained system, giving additional weight to something or someone implies that something or someone else will lose out. The inclusion of equity concerns must therefore always take opportunity costs into consideration.

Use of this guidance is compatible with the use of more quantitative approaches, for example multi criteria decision analysis, to consider equity in evaluations of health interventions through explicit weighing [12,24,54,55]. Another example is the use of so-called equity or distributive weights, which can incorporate special priority to the worst off [14,56]. A third example is a proposal in UK to quantify the value of distributional impact for value based assessments of health technologies [57]. Methods are also being developed to incorporate concern for poverty and financial risk protection in extended cost-effectiveness analysis [58].

Yet, there may always be disagreement about the importance of criteria when setting priorities between health interventions. For this reason, ethicists have stressed the importance of fair processes, which allow key stakeholders to agree on what is legitimate and fair. Key elements of a fair process involves transparency about the grounds for decisions; appeals to rationales that all can accept as relevant to meeting health needs fairly; and procedures for revising decisions in light of challenges to them. Together these elements assure “accountability for reasonableness.” [53]. The GPS-Health should therefore be seen as integral to such legitimate processes.

The GPS-Health feeds into the international debate about social value judgements [59] and the use of different criteria for priority setting in health care, in two ways. First, the present guidance can be considered as complementary to published inventories and mapping of all possibly relevant criteria [12,56] as it is based on intense deliberative processes between experts on the relevance of these criteria and ultimately consensus building [12,60]. Second, many countries around the world mention equity-related criteria in their policy frameworks on the reimbursement of health interventions, but these are sometimes ill-defined and not operationalized [61]. This may be one reason why value judgements other than health maximization are not routinely used in important coverage decisions. The GPS-Health aims to overcome this barrier by offering a ready-to-use checklist to decision-makers. This may help them to make the right decisions on the funding of one intervention and the refusal to fund another.

## Appendix: criteria excluded from the checklist

*Life threatening conditions* is a sub-category of severity and is often a key concern in public debate about priority setting decisions. For example, cancer with metastasis may be life threatening, and interventions that save or extend life for people with such conditions may therefore be assigned very high priority by some. But threat to life cannot be the sole condition of priority. Sometimes interventions with marginal benefits and extremely high costs should not be prioritized. The underlying concern is better captured by the severity criterion combined with effectiveness considerations.

### Age

This guidance does not propose age as an independent criterion. The combination of criteria will indirectly imply some priority to the young. Cost-effectiveness analysis will typically, but not always, favour interventions targeting younger age groups. The productivity criterion also indirectly assigns less value to health gained in old age. Severity and past health loss may often, but not always, favour the young. The more general principle that all persons should have a fair chance to live a long and healthy life will in most cases favour the young, but the justification is not age itself, but who the worst off are in terms of lifetime health. The expert group did not consider whether health gains for the very young should have different marginal weights than for adults [45,62,63].

### Individual responsibility for health

In some instances, potential beneficiaries have an existing health condition clearly associated with past choices or a known risky behaviour with a clear association with future health problems. It could be reasonable to considered whether the intervention has lower value because individuals bears some responsibility and has ability to pay for their own care. Whether governments should also hold individuals responsible for choices that affect health and risk is a controversial question. Health conditions are generally due to a combination of background factors, luck and behaviour, and it is therefore unacceptable to submit patients to differential health care access or financial conditions, unless this respects their own values and preferences [32,64-68].

### Rarity of health condition

Rare conditions, i.e. those with a very low prevalence (<5 cases per 10 000), pose a special challenge for priority setting. Rarity is in itself not ethically relevant. However, rare conditions are often particularly costly to treat because product development costs can only be spread over relative few patients, and interventions may therefore be cost-ineffective. Evidence may also be weak because a small patient population makes it difficult to conduct high quality randomized clinical trials. To some extent the cost problem and the documentation problem are balanced by the fact that the value of treatments for rare conditions is in many cases augmented by other equity concerns, such as concerns for severity and concerns for realization of potentials/fair chances. However, this balancing occurs in far from all rare diseases. To ensure equal access to treatment for patients with rare diseases it is therefore sometimes argued that there should be a higher willingness to pay for treatments for rare conditions than for treatments in general. The problem is that this would give an advantage to patients with rare diseases relative to patients with more common diseases who are costly to treat for other reasons than rarity. Altogether the group did not agree on how rare diseases should be dealt with, but a majority wanted it excluded from the main list of equity criteria on the grounds that rarity is not a value per se.

## Competing interests

The authors declare that they have no competing interests.

## Authors’ contribution

OFN, MJ, RB, DC and TTE had the original idea for this paper. OFN, MJ, RB and DC wrote the first draft, and all authors met three times to discuss, review and comment on the draft. All authors provided written comments on subsequent drafts. All authors read and approved the final manuscript.
